# Phase I Clinical Trial of CVL218, a Novel PARP1/2 Inhibitor, in Patients with Advanced Solid Tumors

**DOI:** 10.1002/mco2.70272

**Published:** 2025-07-09

**Authors:** Zihong Chen, Gang Chen, Yuxiang Ma, Hongyun Zhao, Jianhua Zhan, Yan Huang, Yunpeng Yang, Yuanyuan Zhao, Shaodong Hong, Ting Zhou, Wenfeng Fang, Li Zhang, Yaxiong Zhang

**Affiliations:** ^1^ Department of Medical Oncology State Key Laboratory of Oncology in South China Guangdong Provincial Clinical Research Center for Cancer Collaborative Innovation Center for Cancer Medicine Sun Yat‐sen University Cancer Center Guangzhou China; ^2^ Zhongshan School of Medicine Sun Yat‐sen University Guangzhou China; ^3^ Department of Clinical Research State Key Laboratory of Oncology in South China Guangdong Provincial Clinical Research Center for Cancer Collaborative Innovation Center for Cancer Medicine Sun Yat‐sen University Cancer Center Guangzhou China

**Keywords:** CVL218, cancer, phase I, PARP, pharmacokinetics

## Abstract

CVL218, a novel poly ADP‐ribose polymerase (PARP1/2) inhibitor, has strong PARP1/2 selective inhibitory activity and high oral bioavailability. We aimed to assess the safety and tolerability of CVL218 in patients with pretreated advanced solid tumors. Patients in this phase I dose escalation trial received one dose of CVL218 (50, 100, 200, 350, 500, 600, 700, and 850 mg) twice a day. The safety, tolerability, maximum tolerated dose (MTD), dose‐limiting toxicity (DLT), recommended dose, as well as antitumor activity of CVL218 were evaluated. A total of 26 patients were enrolled in this trial. The most common treatment‐related adverse events were vomiting (76.9%), nausea (76.9%), diarrhea (38.5%), proteinuria (23.1%), and lipase increased (23.1%). DLTs occurred in three patients, one out of six in the 700 mg BID group, and two out of five in the 850 mg BID group, so the MTD was set to 700 mg BID. Overall, the disease control rate (DCR) was 70.8%, while the DCR of patients with high‐level doses (≥700 mg BID) and recommended dose (700 mg BID) were both 100%. CVL218 was generally well tolerated and safe. It showed potential antitumor activity in patients treated with the recommended dose.

## Graphical abstract

CVL218, a PARP1/2 inhibitor, was generally well tolerated and safe in solid tumors. It showed potential antitumor activity in patients treated with the recommended dose (700 mg BID).

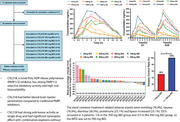

## 1 Introduction

Cells constantly encounter endogenous and exogenous threats that induce DNA damage. If left unrepaired, this damage can cause permanent changes, resulting in alterations to inherited characteristics [1]. The most common and severe forms of DNA damage are DNA strand breaks, which include single‐strand breaks (SSBs) and double‐strand breaks (DSBs) [2]. Typically, cells initiate DNA damage repair immediately after DNA damage occurs. Poly (ADP‐ribose) polymerase (PARP) plays an essential role in DNA damage repair [3]. PARP plays an essential role in this repair process, accurately detecting DNA breaks and binding tightly to the damaged sites. When a single strand is damaged, PARP functions as a molecular sensor and participates in the repair process [3, 4]. For DSBs, the homologous recombination repair (HRR) pathway becomes crucial. Damaged DNA is repaired by utilizing homologous chromatids or sister chromatids as templates to ensure that the broken DNA duplexes are accurately paired and recombined with the template DNA [5, 6, 7].

Genomic instability and the loss of a cell's physiological ability of cells to repair DNA damage are hallmarks of cancer [8]. If the SSB repair mechanism fails, the HRR pathway becomes critical for repairing DSBs, ensuring DNA repair functionality. However, defects in HRR (HRD) can impair DSB repair, leading to genomic instability and an increased risk of malignancy [9]. If both repair mechanisms fail simultaneously, the genome may become excessively damaged, activating intrinsic apoptotic signals in the cells [4]. This indicates that targeting the DNA damage repair system with specific drugs can be effective for patients with defective DNA repair mechanisms [10]. This has spurred the clinical development of PARP inhibitors as novel highly selective antitumor agents in patients with HRD.

Breast cancer susceptibility genes 1 and 2 (BRCA1 and BRCA2) are vital for DNA damage repair and normal cell growth. The proteins encoded by BRCA1 and BRCA2 are involved in DNA end cleavage and repair during HRR [11]. Mutations in BRCA1 and BRCA2 can lead to loss of function, resulting in HRD and subsequent tumorigenesis. BRCA1/2 mutations have been identified in several common cancer types, including ovarian, breast, pancreatic, and prostate cancers [12]. Consequently, several PARP inhibitors have been approved by the United States Food and Drug Administration (US FDA) and the European Medicines Agency for the treatment of various subtypes of advanced breast, ovarian, prostate, and pancreatic cancers [13].

Olaparib was the first and the most well‐known PARP inhibitor to be approved by the US FDA for treatment of multiple types of advanced cancer [14, 15, 16, 17]. However, it has certain limitations, such as low solubility and permeability, high gastrointestinal toxicity and poor stability of the preparation. Previous studies have shown that CVL218, a novel PARP1/2 inhibitor, has strong PARP1/2 selective inhibitory activity and high oral bioavailability [18, 19]. In vitro and in vivo experiments revealed that CVL218 had strong antitumor activity as single drug and had significant synergistic effect with combination regimens without inhibitory activity on the human ether‐a‐go‐go‐related gene channel. The associated toxic reactions were reversible and primarily involved the thymus, bone marrow, kidney and liver. Additionally, another study indicated that CVL218 is widely distributed across various tissues and readily crosses the blood–brain barrier, which is a better result than that of first‐generation PARP inhibitors [19].

Based on the statements above, we conducted a phase I, single‐arm, dose escalation study in patients with advanced solid tumors who had disease progression after previously standard treatment to assess the safety and tolerability of CVL218, as well as the pharmacokinetics (PK) parameters and preliminary antitumor activity.

## 2 Result

### 2.1 Patient Enrollment and Baseline Characteristics

**FIGURE 1:**
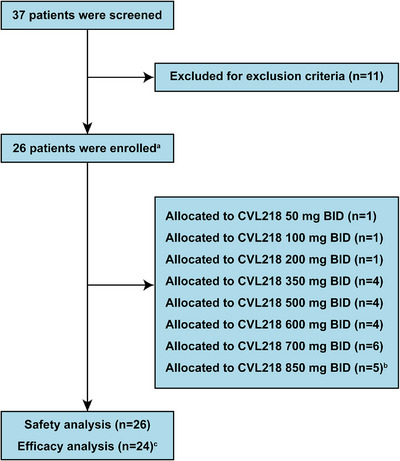
Flow chart of patients enrolled in the study. ^a^All enrolled patients were given a single oral dose for the first day, then multiple dosing (twice a day taking 12 h apart) started continuously after 72 h of single dosing. ^b^Two‐thirds of the subjects developed dose‐limiting toxicity in this cohort, thus discontinuing dose raising. ^c^Two patients fell out during the course of the project without postbaseline tumor assessment.

With a total of 37 subjects screened from July 2019 to July 2021, 26 patients were successfully enrolled in this trial stratified by eight different doses (Figure 1). Two patients in the 850 mg BID cohort developed dose‐limiting toxicity (DLT), leading to discontinuation of dose raising (Table S1). All of the enrolled patients received at least one dose of CVL218 and were included in the safety analysis, while only 24 patients were included in the efficacy analysis due to the absence of postbaseline tumor assessments. Baseline characteristics were summarized in Table 1. The median age of the participants was 55 years, with a majority being men (54%, 14 out of 26) and had an Eastern Cooperative Oncology Group (ECOG) performance status score of 0 (62%, 16 out of 26). Most patients were classified as stage IV (92%, 24 out of 26). The primary tumor types were lung cancer (42%, 11 out of 26) and breast cancer (27%, seven out of 26). Almost half of the patients had received surgery (54%, 14 out of 26) or radiotherapy (46%, 12 out of 26) previously.

**TABLE 1:** Baseline patient demographics and clinical characteristics.

Characteristics	All patients (*N* = 26)
Age, median (range)	55 (29–72)
Gender, *n* (%)	
Male	14 (54)
Female	12 (46)
ECOG, *n* (%)	
0	16 (62)
1	10 (38)
Tumor type^a^, *n* (%)	
Lung cancer	11 (42)
Breast cancer	7 (27)
Other types	8 (31)
Tumor stage, *n* (%)	
III	2 (8)
IV	24 (92)
Prior surgery history, *n* (%)	
Yes	14 (54)
No	12 (46)
Prior radiotherapy history, *n* (%)	
Yes	12 (46)
No	14 (54)

^a^ Other types included adenoid cystic carcinoma (*n* = 2), cholangiocarcinoma (*n* = 1), gallbladder carcinoma (*n* = 1), ovarian cancer (*n* = 1), prostate cancer (*n* = 1), uterine leiomyosarcoma (*n* = 1), and nasopharyngeal carcinoma (*n* = 1).

### 2.2 Safety and Tolerability

All of the patients experienced at least one treatment‐related adverse events (TRAEs) (Table 2). The most frequently reported (≥20%) TRAEs of any grade included vomiting (76.9%), nausea (76.9%), diarrhea (38.5%), proteinuria (23.1%), and lipase increased (23.1%). Most AEs were grade 1–2 and easily managed. Only four patients (15.4%, 850 mg BID, *n* = 2; 600 mg BID, *n* = 1; 500 mg BID, *n* = 1) experienced ≥grade 3 TRAEs, including blood creatinine increased (11.5%), blood urea increased (3.8%), lipase increased (3.8%), and abdominal pain (3.8%) during treatment. A total of three serious adverse events occurred, including one (ascites) in 700 mg BID dose group and two (one aspartate aminotransferase elevation and one radiation encephalopathy) in 850 mg BID dose group. Regarding DLTs (Table S1), one of six patients who were eligible for DLT assessment experienced a DLT (renal failure) at 700 mg BID, while two of five patients experienced DLTs (≥grade 3 increased creatinine) at 850 mg BID. Thus, the maximum tolerated dose (MTD) was 700 mg BID, and the recommended dose for further study of CVL218 was determined to be 700 mg BID.

**TABLE 2:** Treatment‐related adverse events reported in all patients.

System organ class Preferred Term	50 mg BID (*N* = 1)		100 mg BID (*N* = 1)		200 mg BID (*N* = 1)		350 mg BID (*N* = 4)		500 mg BID (*N* = 4)		600 mg BID (*N* = 4)		700 mg BID (*N* = 6)		850 mg BID (*N* = 5)		All (*N* = 26)	
*n* (%)	ALL	≥G3	ALL	≥G3	ALL	≥G3	ALL	≥G3	ALL	≥G3	ALL	≥G3	ALL	≥G3	ALL	≥G3	ALL	≥G3
Any AE related to study drug	1 (100)	0	1 (100)	0	1 (100)	0	4 (100)	0	4 (100)	1 (25.0)	4 (100)	1 (25.0)	6 (100)	0	5 (100)	2 (40.0)	26 (100)	4 (15.4)
Metabolic disorders	0	0	0	0	0	0	0	0	2 (50.0)	0	1 (25.0)	0	1 (16.7)	0	2 (40.0)	0	6 (23.1)	0
Hypoalbuminemia	0	0	0	0	0	0	0	0	0	0	1 (25.0)	0	1 (16.7)	0	0	0	2 (7.7)	0
Decreased appetite	0	0	0	0	0	0	0	0	2 (50.0)	0	0	0	1 (16.7)	0	2 (40.0)	0	5 (19.2)	0
General disorders	0	0	0	0	1 (100)	0	1 (25.0)	0	1 (25.0)	0	1 (25.0)	0	0	0	0	0	4 (15.4)	0
Asthenia	0	0	0	0	0	0	0	0	1 (25.0)	0	1 (25.0)	0	0	0	0	0	2 (7.7)	0
Chest discomfort	0	0	0	0	0	0	1 (25.0)	0	0	0	1 (25.0)	0	0	0	0	0	2 (7.7)	0
Infusion site pain	0	0	0	0	1 (100)	0	0	0	0	0	0	0	0	0	0	0	1 (3.8)	0
Musculoskeletal disorders	1 (100)	0	0	0	0	0	0	0	0	0	0	0	0	0	0	0	1 (3.8)	0
Musculoskeletal discomfort	1 (100)	0	0	0	0	0	0	0	0	0	0	0	0	0	0	0	1 (3.8)	0
Limb discomfort	1 (100)	0	0	0	0	0	0	0	0	0	0	0	0	0	0	0	1 (3.8)	0
Nervous system disorders	0	0	1 (100)	0	0	0	1 (25.0)	0	0	0	0	0	2 (33.3)	0	0	0	4 (15.4)	0
Dizziness	0	0	0	0	0	0	1 (25.0)	0	0	0	0	0	2 (33.3)	0	0	0	3 (11.5)	0
Headache	0	0	1 (100)	0	0	0	0	0	0	0	0	0	1 (16.7)	0	0	0	2 (7.7)	0
Cardiac disorders	0	0	0	0	0	0	0	0	0	0	1 (25.0)	0	0	0	0	0	1 (3.8)	0
Palpitations	0	0	0	0	0	0	0	0	0	0	1 (25.0)	0	0	0	0	0	1 (3.8)	0
Reproductive disorders	0	0	0	0	0	0	0	0	0	0	0	0	1 (16.7)	0	0	0	1 (3.8)	0
Erectile dysfunction	0	0	0	0	0	0	0	0	0	0	0	0	1 (16.7)	0	0	0	1 (3.8)	0
Skin disorders	0	0	0	0	0	0	0	0	1 (25.0)	0	2 (50.0)	0	0	0	0	0	3 (11.5)	0
Rash	0	0	0	0	0	0	0	0	1 (25.0)	0	2 (50.0)	0	0	0	0	0	3 (11.5)	0
Renal & urinary disorders	0	0	1 (100)	0	0	0	0	0	1 (25.0)	0	1 (25.0)	0	3 (50.0)	0	1 (20.0)	0	7 (26.9)	0
Renal failure	0	0	0	0	0	0	0	0	0	0	0	0	3 (50.0)	0	0	0	3 (11.5)	0
Proteinuria	0	0	1 (100)	0	0	0	0	0	1 (25.0)	0	1 (25.0)	0	2 (33.3)	0	1 (20.0)	0	6 (23.1)	0
Gastrointestinal disorders	1 (100)	0	1 (100)	0	0	0	4 (100)	0	4 (100)	0	3 (75)	0	6 (100)	0	5 (100)	1 (20)	24 (92.3)	1 (3.8)
Abdominal pain upper	0	0	0	0	0	0	0	0	0	0	0	0	0	0	1 (20.0)	0	1 (3.8)	0
Constipation	0	0	0	0	0	0	0	0	0	0	0	0	0	0	2 (40.0)	0	2 (7.7)	0
Vomiting	0	0	0	0	0	0	3 (75)	0	3 (75)	0	3 (75)	0	6 (100)	0	5 (100)	0	20 (76.9)	0
Nausea	0	0	1 (100)	0	0	0	3 (75)	0	3 (75)	0	2 (50)	0	6 (100)	0	5 (100)	0	20 (76.9)	0
Gingival bleeding	1 (100)	0	0	0	0	0	0	0	0	0	0	0	0	0	0	0	1 (3.8)	0
Diarrhea	0	0	0	0	0	0	1 (25)	0	1 (25)	0	2 (50)	0	4 (66.7)	0	2 (40.0)	0	10 (38.5)	0
Abdominal pain	0	0	0	0	0	0	0	0	0	0	0	0	0	0	1 (20.0)	1 (20)	1 (3.8)	1 (3.8)
Abdominal distension	0	0	0	0	0	0	0	0	1 (25)	0	0	0	0	0	0	0	1 (3.8)	0
Blood system disorders	0	0	0	0	0	0	0	0	0	0	0	0	0	0	2 (40.0)	0	2 (7.7)	0
Anemia	0	0	0	0	0	0	0	0	0	0	0	0	0	0	2 (40.0)	0	2 (7.7)	0
Laboratory test disorders	0	0	0	0	1 (100)	0	1 (25)	0	3 (75)	1 (25)	3 (75)	1 (25)	2 (33.3)	0	5 (100)	2 (40.0)	15 (57.7)	4 (15.4)
ALT increased	0	0	0	0	0	0	1 (25)	0	2 (50)	0	0	0	1 (16.7)	0	0	0	4 (15.4)	0
Neutrophil count increased	0	0	0	0	0	0	0	0	1 (25)	0	0	0	0	0	1 (20.0)	0	2 (7.7)	0
Neutrophil count decreased	0	0	0	0	0	0	0	0	0	0	0	0	0	0	1 (20.0)	0	1 (3.8)	0
AST increased	0	0	0	0	0	0	1 (25)	0	0	0	0	0	1 (16.7)	0	0	0	2 (7.7)	0
Urinary occult blood positive	0	0	0	0	0	0	0	0	0	0	0	0	0	0	1 (20.0)	0	1 (3.8)	0
Protein urine present	0	0	0	0	0	0	0	0	0	0	0	0	0	0	1 (20.0)	0	1 (3.8)	0
Amylase increased	0	0	0	0	0	0	0	0	1 (25)	0	1 (25)	0	0	0	0	0	2 (7.7)	0
WBC count increased	0	0	0	0	0	0	0	0	1 (25)	0	0	0	0	0	1 (20.0)	0	2 (7.7)	0
WBC count decreased	0	0	0	0	0	0	0	0	0	0	0	0	0	0	1 (20.0)	0	1 (3.8)	0
Granulocyte count increased	0	0	0	0	0	0	0	0	0	0	0	0	0	0	1 (20.0)	0	1 (3.8)	0
Lipase increased	0	0	0	0	1 (100)	0	0	0	1 (25)	1 (25)	2 (50)	0	0	0	2 (40.0)	0	6 (23.1)	1 (3.8)
Blood urea increased	0	0	0	0	0	0	0	0	0	0	1 (25)	1 (25)	0	0	0	0	1 (3.8)	1 (3.8)
Blood triglycerides increased	0	0	0	0	0	0	0	0	0	0	0	0	0	0	1 (20.0)	0	1 (3.8)	0
Blood creatinine increased	0	0	0	0	0	0	0	0	0	0	1 (25)	1 (25)	0	0	4 (80.0)	2 (40.0)	5 (19.2)	3 (11.5)

Abbreviations: ALT, alanine aminotransferase; AST, aspartate aminotransferase; WBC, white blood cell.

### 2.3 Pharmacokinetics

**FIGURE 2:**
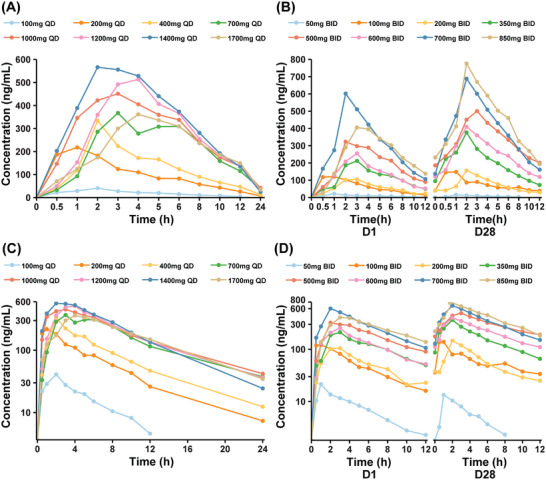
Concentration–time profiles and Log concentration–time profiles of plasma CVL218 levels by cohort after a single dose (cycle 0 Day 1) and multiple doses (cycle 1 Day 28). Concentration–time profiles of plasma CVL218 levels by cohort after (A) a single dose (cycle 0 Day 1) and (B) multiple doses (cycle 1 Day 1 and cycle 1 Day 28). Log concentration–time profiles of plasma CVL218 levels by cohort after (C) a single dose (cycle 0 Day 1) and (D) multiple doses (cycle 1 Day 1 and cycle 1 Day 28).

The PK parameters of CVL218 were evaluated after single‐ and multiple‐dose administration. Prior to initiating a continuous daily treatment cycle, patients participated in a single‐dose PK treatment period with a 72‐h washout. Figure 2A,B shows plasma CVL218 concentration–time plots for each cohort on Day 1 after a single dose as well as on Day 1 and Day 28 after multiple doses, respectively. The semi‐log plot was also provided in Figure 2C,D, respectively. The PK parameter and steady‐state PK parameters summary is provided in Tables S2 and S3. CVL218 was rapidly absorbed after oral administration with a median time to reach peak concentration (*C*_max_) (*T*_max_) of 1–4 h for a single dose and 1–3 h for multiple doses. The observed mean half‐life ranged from 3.6 to 6.3 h for a single oral dose and from 3.9 to 5.4 h for multiple oral doses.

*C*_max_ and the area under the plasma concentration time curve from 0 to last measurable concentration (AUC_0–_ *_t_*) for different dose groups on C0D1 and C1D1 are presented in Figure S1. Overall, both *C*_max_ and AUC_0–_ *_t_* showed an increasing trend with dose escalation, despite some individual variability. The recommended dose group exhibited the highest *C*_max_ and AUC_0–_ *_t_* on C0D1 compared with all other dose groups. Similarly, on C1D1, the recommended dose group also had the highest *C*_max_. A statistically significant difference in *C*_max_ was observed between the 700 mg BID and 350 mg BID groups, while no significant differences were found among the other dose groups. And the dose linearity evaluation was listed in Figure S2. Moreover, the SD group exhibited higher *C*_max_ and AUC_0–_ *_t_* than the progressive disease (PD) group (Figure S3).

### 2.4 Antitumor Activities

**FIGURE 3:**
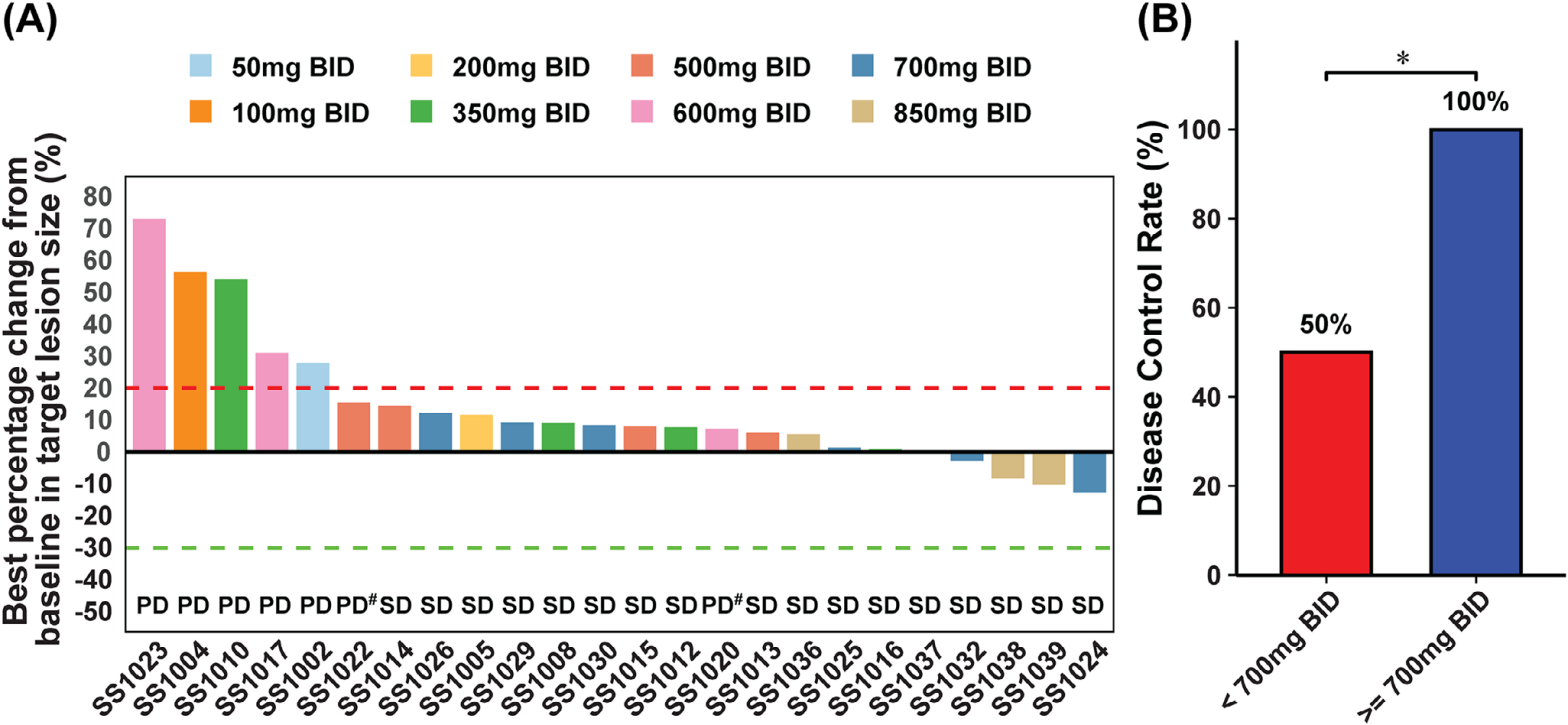
Efficacy of CVL218. (A) Waterfall plot of tumor response. #Two patients (SS1020 and SS1022) were evaluated as PD due to new lesions, although the evaluation of target lesion was SD. (B) Disease control rate between dose groups.*p < 0.05. *Abbreviations*: SD, stable disease; PD, progressive disease.

We also evaluated the preliminary efficacy of CVL218. Among the 26 subjects, two subjects dropped out during the course of the project without imaging evaluation data. The remaining 24 subjects were included in efficacy analysis set and none of them achieved complete response (CR) or partial response (PR). The specific DCR rates for each dose group are detailed in Table 3. Figure 3A illustrates the waterfall plot of tumor response across the different doses. Overall, 17 out of 24 patients achieved stable disease (SD), leading to a disease control rate (DCR) of 70.8%. All patients in the high‐dose group (≥700 mg BID, *n* = 10) and those receiving the recommended dose (700 mg BID, *n* = 6) achieved SD, resulting in a DCR of 100% for both groups. In contrast, patients in low‐level dose group (<700 mg BID, *n* = 14) had a DCR of only 50%, indicating a significant difference compared with the high‐level dose group (Figure 3B). Table S4 revealed the tumor response according to tumor types. The DCR was 60% for lung cancer, 71.4% for breast cancer, and 85.7% for other cancers.

**TABLE 3:** Tumor response according to different doses.

	50 mg BID (*N* = 1)	100 mg BID (*N* = 1)	200 mg BID (*N* = 1)	350 mg BID (*N* = 4)	500 mg BID (*N* = 4)	600 mg BID (*N* = 3)	700 mg BID (*N* = 6)	850 mg BID (*N* = 4)	Overall (*N* = 24)
BOR, *n* (%)									
CR	0	0	0	0	0	0	0	0	0
PR	0	0	0	0	0	0	0	0	0
SD	0	0	1 (100)	3 (75.0)	3 (75.0)	0	6 (100)	4 (100)	17 (70.8)
PD	1 (100)	1 (100)	0	1 (25.0)	1 (25.0)	3 (100)	0	0	7 (29.2)
ORR, *n* (%) (95%CI)	0 (0, 97.5)	0 (0, 97.5)	0 (0, 97.5)	0 (0, 60.2)	0 (0, 60.2)	0 (0, 70.8)	0 (0, 45.9)	0 (0, 60.2)	0 (0, 14.3)
DCR, *n* (%) (95%CI)	0 (0, 97.50)	0 (0, 97.50)	1 (100) (2.5, 100.0)	3 (75.0) (19.4, 99.4)	3 (75.0) (19.4, 99.4)	0 (0, 70.8)	6 (100) (54.1, 100.0)	4 (100) (39.8, 100.0)	17 (70.8) (48.9, 87.4)
mPFS (months)	1.05	1.05	1.97	2.34	3.27	1.02	2.27	2.35	1.89

Abbreviations: BOR, best overall response; CR, complete response; DCR, disease control rate; mPFS, median progression‐free survival; ORR, objective response rate; PD, progressive disease; PR, partial response; SD, stable disease.

Among the 26 subjects enrolled in our study, progression‐free survival (PFS) events occurred in 24 patients and the median PFS (mPFS) was 1.89 months. In the subgroup analysis, the mPFS was 2.79 months for 17 patients with SD and 1.05 months for 7 patients whose best overall response was PD. For dose levels, the mPFS was 2.35 months for 10 patients on high‐level doses (≥700 mg BID) and 1.80 months for 14 patients on low‐level doses (<700 mg BID), as detailed in Table 3. Among the patients with lung cancer, the mPFS was 2.12 months, while for 7 breast cancer patients, it was 1.71 months. The remaining seven patients with other cancer types had a mPFS of 1.91 months (Table S4).

## 3 Discussion

DNA lesions may occur spontaneously or be induced by external factors. Timely repair is required to ensure normal function. DNA damage is primarily repaired through one or more of the following six major repair pathways: direct repair; base excision repair (BER); nucleotide excision repair; mismatch repair; HRR or non‐homologous end joining pathways [8]. PARP proteins detect DNA damage in form of SSB. Once PARP has bound to the damaged DNA, it changes its structure and recruits DNA repair proteins to repair the SSB through BER [20]. Nevertheless, DNA damage can be exploited. PARP inhibitors bind to PARP and block BER. In this scenario, DNA SSB cannot be repaired and remains during replication. This SSB can result in a more severe DNA DSB [8]. In normal cells, DNA DSB can be repaired by HRR. Conversely, in patients with BRCA1/2 mutations, there is a “synthetic lethality” effect [21]. With the use of PARP inhibitors, SSBs cannot be repaired, leading to DSB that BRCA1/2 mutation carriers cannot repair through HRR. The DNA DSB remains unrepaired and it is likely to cause genomic instability which can end up in apoptosis. This is also the reason that PARP inhibitors are particularly effective in patients with BRCA1/2 mutation.

The most common adverse reactions associated with PARP inhibitors are hematologic, primarily presenting as anemia, neutropenia, and thrombocytopenia. Gastrointestinal side effects characterized by nausea, vomiting, and diarrhea was also prevalent. Notably, the majority of grade ≥3 AEs are hematological, which are also the leading causes of dose reduction, interruption, or discontinuation of treatment [22]. The overall incidence of grade ≥3 AEs for olaparib was 36–40%. The rate of dose reduction rate due to AEs was approximately 25–29%, while treatment discontinuation rates was about 6–17% [14, 17, 23]. In our study, however, only four patients experienced grade ≥3 AEs, resulting in an overall incidence of grade ≥3 AEs of 15.4%, with half of these occurring in the 800 mg BID dose group.

The incidence of anemia among hematologic AEs with olaparib ranges from 21 to 46%, with the occurrence of grade ≥3 anemia reported at approximately 5.1–22% [14, 16, 17, 24]. In comparison, niraparib reported an overall incidence of anemia between 49.7 and 53.1%, with grade ≥3 incidence rates of 14.7–22.5% [25, 26, 27]. In our study, we observed the occurrence of anemia only in the dose of 850 mg BID, with an overall incidence of 7.7%. None of the anemias were of grade 3 or more severe. This may be attributable to the enhanced selectivity of CVL218. The higher rates of severe adverse reactions associated with conventional PARP inhibitors necessitate dose reductions or treatment discontinuations in these patients, potentially impacting therapeutic efficacy. In this regard, CVL218 demonstrates a distinct advantage over traditional PARP inhibitors. The toxicity of CVL218 is primarily manifested in gastrointestinal reactions, including nausea, vomiting, diarrhea, and so on, as well as increased creatinine and proteinuria. Gastrointestinal reactions were present in 92.3% of patients, but the overall severity was mild, and only one patient (3.8%) experienced grade ≥3 gastrointestinal AEs, which is comparable to that for olaparib (4.3%).

It is crucial to remain vigilant about long‐term adverse effects associated with conventional PARP inhibitors, such as myelodysplastic syndromes (MDS) and acute myeloid leukemia (AML). The incidence of MDS/AML attributable to olaparib was approximately 1% [28, 29]. Olaparib is associated with a 158% increased risk of MDS/AML compared with placebo, while Niraparib is linked to a 48% increase in risk [23, 29]. Real‐world pharmacovigilance data suggest a strong correlation between anemia and the diagnosis of MDS and AML, highlighting the need for heightened awareness of these conditions in anemic patients [29]. Currently, CVL218 is still under clinical investigation, and long‐term adverse reactions such as MDS/AML have not been observed. Other adverse effects, including reproductive toxicity or the risk of secondary malignancies, are still being monitored in follow‐up studies.

In this dose‐escalation phase I clinical study, we found that CVL218 demonstrated well performance in safety from 50 mg BID to 700 mg BID. And the recommended dose was 700 mg BID. PK analysis indicated that CVL218 is rapidly absorbed after oral administration, with a median *T*_max_ of 1–4 h for single dose and 1–3 h for multiple doses. At the recommended dose of 700 mg BID, the median *T*_max_ was 2 h, compared with 1.5 h for olaparib at a recommended dose of 300 mg BID [30]. The exposure *C*_max_ of CVL218 increased with dose escalation, while the AUC did not increase proportionately with the dose. This can be attributed to the saturation of the target at the 700 mg BID dose. The binding sites for the drug reached their maximum capacity, preventing further increases in bioavailability and efficacy. Additionally, at the 850 mg BID dose, one patient experienced serious vomiting during treatment, resulting in reduced exposure and affecting the AUC values.

Although the safety of CVL218 was controllable, the efficacy in this study was unsatisfactory. On the one hand, this was a dose‐escalation study, with a total of eight dose groups ranging from 50 mg BID to 850 mg BID. For the low‐dose group, the efficacy was relatively poor. Most of these patients were evaluated as PD. For patients in the high‐dose group, that is, patients received 700 mg BID or 850 mg BID, the efficacy was much better. The DCR of high‐dose group was 100%. However, even in the high‐dose groups, no PR or CR were observed. This may be due to the fact that these patients had previously undergone multiple lines of treatment for advanced tumors, resulting in a heavy disease burden. Furthermore, CVL218, as a PARP inhibitor, may exhibit better efficacy in patients with BRCA mutations. However, patients in this study were not specifically screened out for BRCA mutation in this study, which also led to the less impressive efficacy of CVL218 in this study. We reviewed the clinical records of the patients and found that the vast majority of patients had not undergone genetic testing. Only two patients did a genetic test but none of them were detected with a BRAC mutation.

Thus, future clinical research should investigate the efficacy of CVL 218 at the dose of 700 mg BID furtherly. Taking the PK results into account, subsequent studies should focus on patients with BRCA mutations or those whose homologous recombination deficiency (HRD) biomarkers across various solid tumors (advanced or metastatic malignancies, primarily including breast cancer, prostate cancer, lung cancer, bladder cancer, gastric cancer, colorectal cancer, biliary tract tumors, pancreatic cancer, melanoma, glioma, etc.) were positive. In future dose expansion study, the efficacy of CVL218, including objective response rates (ORRs), DCRs, and progression‐free survival, should become the main target.

In conclusion, CVL218 was generally well tolerated and safe in this phase I clinical trial. It showed potential antitumor activity in patients treated with the recommended dose. The results support the ongoing evaluation of the clinical activity of CVL218 in the treatment of cancer patients.

## 4 Materials and Methods

### 4.1 Study Design and Eligibility Criteria

This study was a single‐arm, dose‐escalation phase I trial conducted in Sun Yat‐sen University Cancer Center in China. This study assessed CVL218 in patients with histologically or cytologically confirmed advanced solid tumors who were in PD after standard therapeutic regimens or lacked standard treatment options. Inclusion criteria included: age 18–75 years; with at least one measurable and evaluable target lesion per Response Evaluation Criteria in Solid Tumors 1.1 (RECIST 1.1); ECOG performance status of 0 or 1; adequate hematological, renal, cardiac, and hepatic functions and a life expectancy ≥12 weeks. Key exclusion criteria included prior usage of PARP inhibitors; allergic to the research drug or its components; with active, unstable systemic diseases or active, uncontrolled infection; a history of other malignancies within 5 years, and pregnant or lactating women.

### 4.2 Patient Treatment

CVL218 was administered orally, and this dose‐escalation trial included nine dose levels: 100 mg QD (50 mg BID), 200 mg QD (100 mg BID), 400 mg QD (200 mg BID), 700 mg QD (350 mg BID), 1000 mg QD (500 mg BID), 1200 mg QD (600 mg BID), 1400 mg QD (700 mg BID), 1700 mg QD (850 mg BID), and 2000 mg QD (1000 mg BID). Patients enrolled in the study received a single oral dose on the first day, followed by continuous multiple dosing (twice a day taking 12 h apart) after 72 h, with the treatment cycle lasting 4 weeks. The period during which patients received a single dose is designated as cycle 0 (C0), lasting 72 h. After this period, patients begin multiple dosing, referred to as cycle 1 (C1). An initial accelerated titration design was employed for the three lowest dose groups, and the “3+3” design was applied starting with the 300 mg BID cohort.

### 4.3 Blood Collection

A clinical PK study of CVL218 was conducted, involving blood samples collection during single‐dose administration (before treatment and 0.5, 1, 2, 3, 4, 5, 6, 8, 10, 12, and 24 h after treatment on C0D1) and multiple dosing (before treatment and 0.5, 1, 2, 3, 4, 5, 6, 8, 10, and 12 h after treatment on C1D1; before treatment on C1D8, C1D15, and C1D22; before treatment and 0.5, 1, 2, 3, 4, 5, 6, 8, 10, and 12 h after treatment on C1D28)

### 4.4 Study Objectives

The primary objectives were to assess safety and tolerability as well as to determine the MTD, DLT, and dose recommended for further study. DLT was defined as any of the following events occurring during cycle 0 (including 72 h wash‐out period after a single dose) and cycle 1 per Common Terminology Criteria for Adverse Events (CTCAE) version 5.0: grade 4 anemia; grade 4 neutropenia; grade 3 neutropenia with fever ≥38.5°C; grade 4 thrombocytopenia; grade 3 thrombocytopenia associated with bleeding; grade ≥2 neurotoxicity; any other grade ≥3 nonhematological toxicities (excluding alopecia). The secondary objectives were to assess the PK and preliminary efficacy of CVL218.

### 4.5 Study Assessments

Single‐dose and multiple‐dose PK parameters included *C*_max_, *T*_max_, elimination half‐life (*T*_1/2_), AUC_0–_ *_t_*, AUC_0–inf_, oral clearance (CL/F), and apparent volume of distribution (*V*_z_/*F*) for CVL218. Tumor response was assessed per RECIST 1.1. ORR was defined as proportion of patients with a CR or PR on at least one visit, while DCR was defined as proportion of patients with a CR or PR or SD. PFS was defined as the time from the first dose of either drug to investigator assessed radiologic PD or to death of any cause. PFS would be censored at the time of last tumor assessment.

### 4.6 Statistical Analysis

Subjects who received at least one dose of CVL218 with postadministration safety records were included in safety analysis set. Subjects who received at least one dose of CVL218 with at least one evaluable PK parameter were included in PK analysis set. Per‐protocol set were used for efficacy analysis. Descriptive statistics of safety were presented per CTCAE v5.0. The PK parameters *T*_max_ and *T*_1/2_ were shown as the median (min, max) and mean ± SD, respectively. A two‐sided *p* < 0.05 was considered to indicate statistical significance. Statistical analysis was performed by using GraphPad prism 8.0.2 and R‐4.3.3.

## Author Contributions

Zihong Chen: methodology, visualization, investigation, writing—original draft, writing—review and editing. Gang Chen, Yuxiang Ma, Hongyun Zhao, and Jianhua Zhan: methodology, visualization, investigation, writing—original draft, writing—review and editing. Yan Huang and Yunpeng Yang: conceptualization, methodology, visualization, resources, investigation, writing—review and editing. Yuanyuan Zhao, Shaodong Hong, and Ting Zhou: conceptualization, methodology, visualization, resources, supervision, writing—review and editing. Wenfeng Fang: conceptualization, methodology, visualization, resources, investigation, supervision, writing—review and editing. Li Zhang: conceptualization, methodology, visualization, investigation, resources, writing—review and editing. Yaxiong Zhang: conceptualization, methodology, data curation, visualization, investigation, resources, writing—original draft, writing—review and editing. All authors have full access to the study data and take responsibility for the integrity and accuracy of the data analysis. All authors have read and approved the final manuscript.

## Conflicts of Interest

Compound is patent, but has no potential relevant financial or nonfinancial interests to disclose. The other authors declare no conflicts of interest.

## Ethics Statement

The trial adhered to ethical guidelines and received approval by the Ethics Committee of Sun Yat‐sen University Cancer Center (A2019‐007‐01) and conducted in accordance with the Declaration of Helsinki and Good Clinical Practice. All participants provided their written informed consent prior to inclusion, and the study has been registered on “www.chinadrugtrials.org.cn” under the identifier CTR20190906.

## Supporting information



Supporting File 1: mco270272‐sup‐0001‐SuppMat.docx

## Data Availability

The datasets used and/or analyzed during the current study are available from the corresponding author upon reasonable request.
