# Supplementary material for: Phase I Clinical Trial of CVL218, a Novel PARP1/2 Inhibitor, in Patients with Advanced Solid Tumors
Source: MedComm (2020). 2025 Jul 9;6(7):e70272. doi: 10.1002/mco2.70272 (PMC12238723; doi:10.1002/mco2.70272)
Supplement: Supplementary file 1 — Supporting File 1: mco270272‐sup‐0001‐SuppMat.docx [file MCO2-6-e70272-s001.docx]

**Supplementary Materials for**

**Phase I clinical trial of CVL218, a novel PARP1/2 inhibitor, in patients with advanced solid tumors**

Zihong Chen^1,2,#^, Gang Chen^1,#^, Yuxiang Ma^3,#^, Hongyun Zhao^3,#^ , Jianhua Zhan^1,#^ , Yan Huang^1^, Yunpeng Yang^1^, Yuanyuan Zhao^1^, Shaodong Hong^1^, Ting Zhou^1^, Wenfeng Fang^1,*^, Li Zhang^1,*^, Yaxiong Zhang^1, *^,

^#^ Zihong Chen, Gang Chen, Yuxiang Ma, Hongyun Zhao, Jianhua Zhan contributed equally to this work.

^1^ Department of Medical Oncology, State Key Laboratory of Oncology in South China, Guangdong Provincial Clinical Research Center for Cancer, Collaborative Innovation Center for Cancer Medicine, Sun Yat-sen University Cancer Center; Guangzhou, China;

^2^ Zhongshan School of Medicine, Sun Yat-sen University, Guangzhou, China;

^3^ Department of Clinical Research, State Key Laboratory of Oncology in South China, Guangdong Provincial Clinical Research Center for Cancer, Collaborative Innovation Center for Cancer Medicine, Sun Yat-sen University Cancer Center; Guangzhou, China.

***Correspondence to:**

Yaxiong Zhang (Email: zhangyx@sysucc.org.cn).

Li Zhang (Email: zhangli6@mail.sysu.edu.cn).

Wenfeng Fang, (Email: fangwf@sysucc.org.cn)

**Supplementary Content**

**Figure S1 Pharmacokinetics among dose groups.**

**Figure S2 Dose linearity evaluation.**

**Figure S3 Pharmacokinetics and efficacy.**

**Table S1 DLTs reported in all patients.**

**Table S2 PK parameters of plasma CVL218 by cohort after a single dose (Cycle 0 Day 1).**

**Table S3 PK parameters of plasma CVL218 by cohort after multiple doses (Cycle 1 Day 28).**

**Table S4 Tumor response according to tumor types.**

**Figure S1 Pharmacokinetics among dose groups.** (A) C_max_ and (B) AUC_0-t_ among different dose groups on C0D1. (C) C_max_ and (B) AUC_0-t_ among different dose groups on C1D1. * *p* < 0.05.

Abbreviations: C_max_, peak concentration; AUC_0-t_, the area under the plasma concentration time curve from 0 to last measurable concentration.


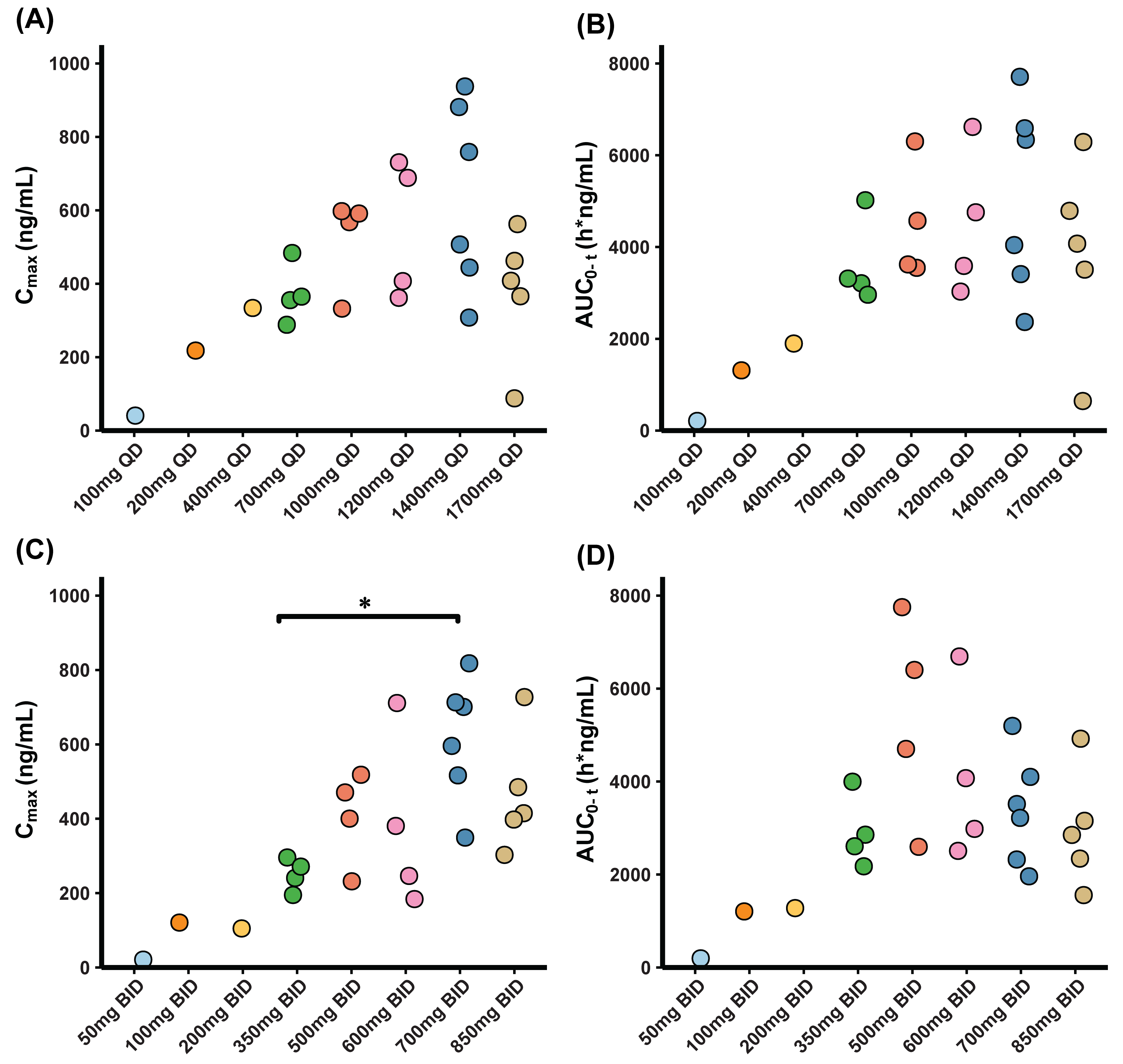


**Figure S2 Dose linearity evaluation.** Dose linearity evaluation between dose and **(A)** C_max_ and (B) AUC0-t on C0D1. Dose linearity evaluation between dose and **(C)** C_max_ and (D) AUC0-t on C1D1.

Abbreviations: C_max_, peak concentration; AUC_0-t_, the area under the plasma concentration time curve from 0 to last measurable concentration.

**
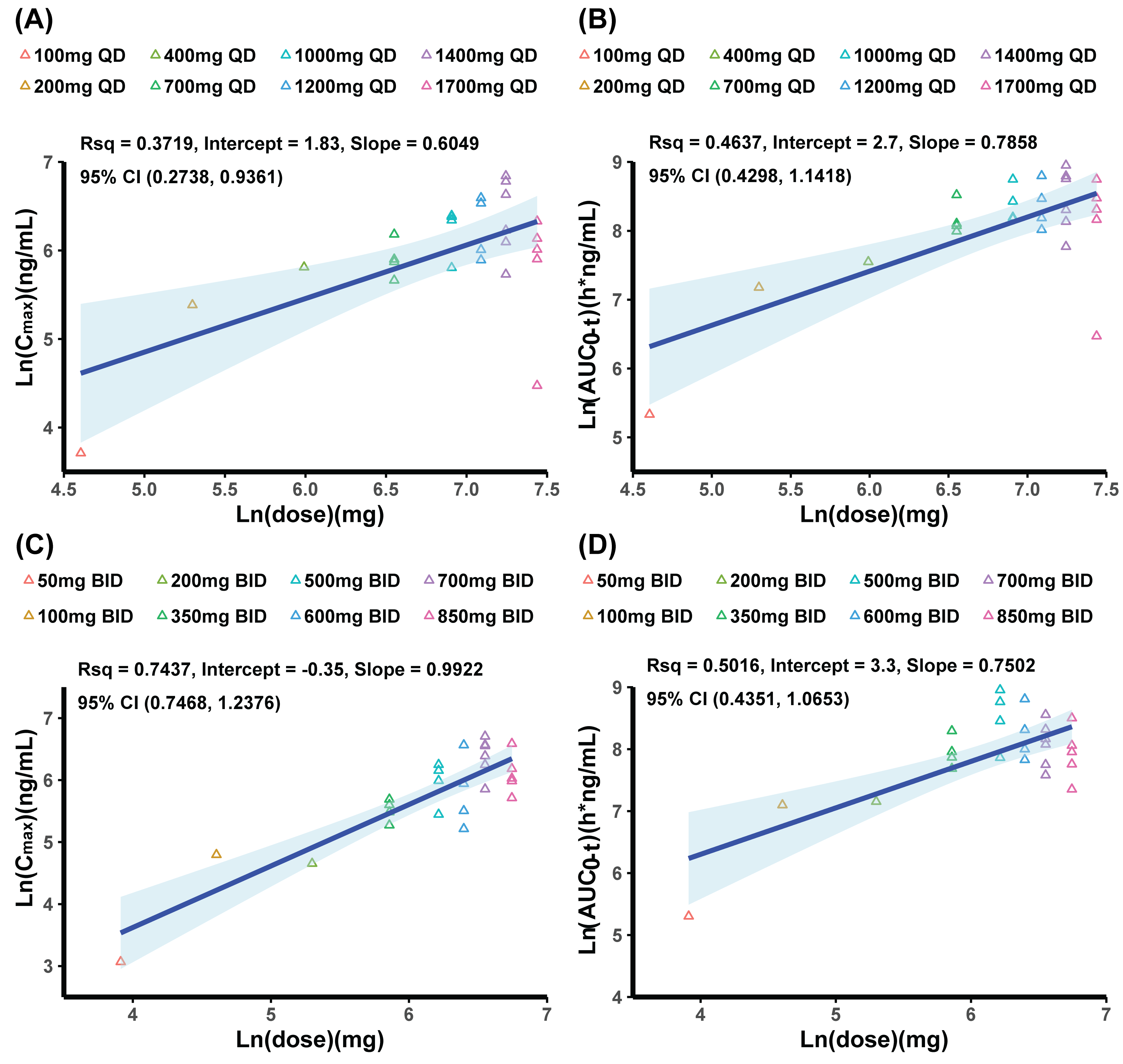
**

**Figure S3 Evaluation of pharmacokinetics and efficacy.** (A) C_max_ and (B) AUC_0-t_ levels on C0D1 between BOR groups. (C) C_max_ and (D) AUC_0-t_ levels on C1D1 between BOR groups. Boxplot center line, median; box limits, upper and lower quartiles; whiskers, 1.5× interquartile range.

Abbreviations: C_max_, peak concentration; AUC_0-t_, the area under the plasma concentration time curve from 0 to last measurable concentration; BOR, best of response.


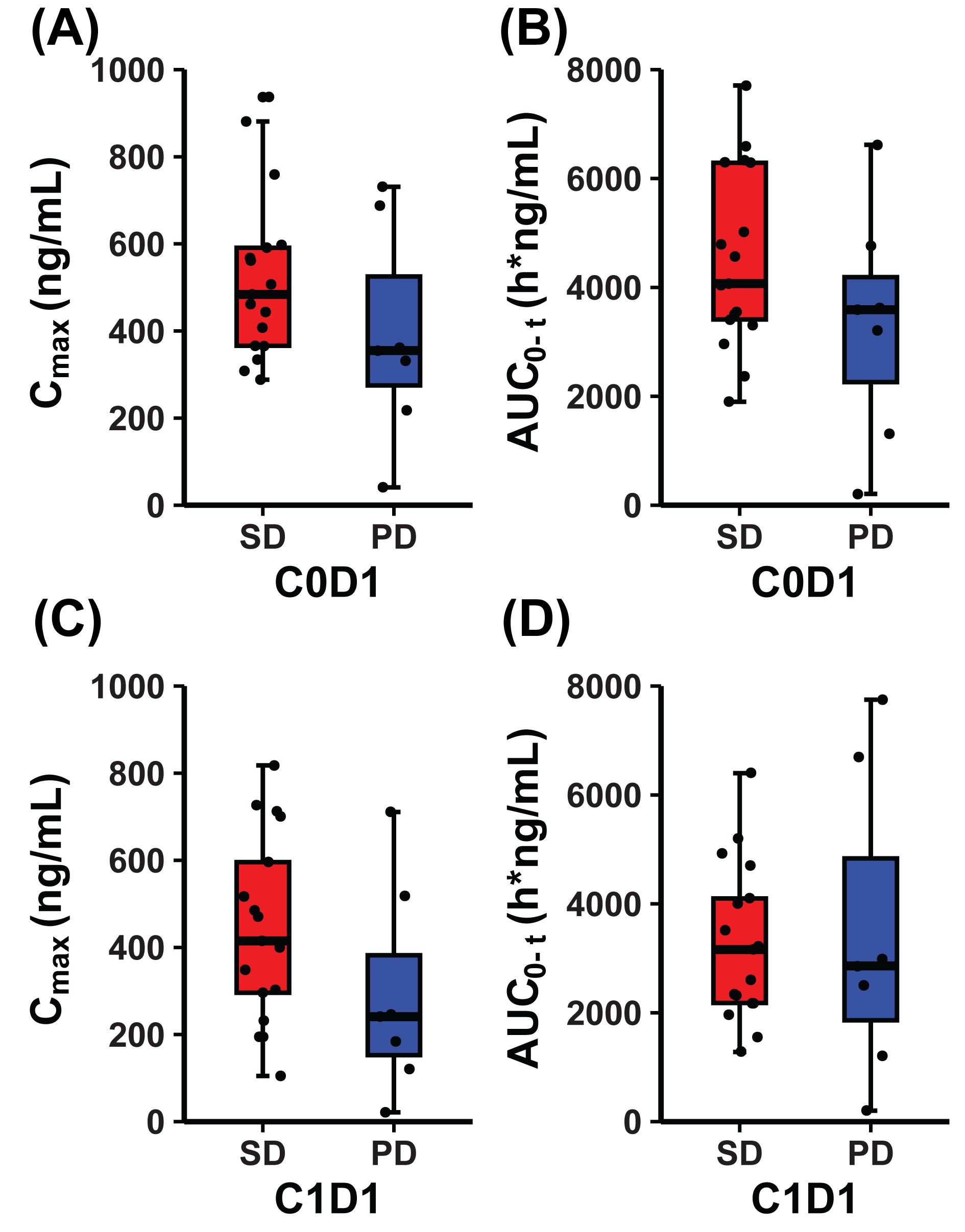


**Table S1 DLTs reported in all patients.**

| **Events**  n (%) | **50mg BID (N=1)** | **100mg BID (N=1)** | **200mg BID (N=1)** | **350mg BID (N=4)** | **500mg BID (N=4)** | **600mg BID (N=4)** | **700mg BID (N=6)** | **850mg BID (N=5)** | **Overall (N=26)** |
| --- | --- | --- | --- | --- | --- | --- | --- | --- | --- |
| **Any DLT** | 0 | 0 | 0 | 0 | 0 | 0 | 1 (16.7) | 2 (40.0) | 3 (11.5) |
| **Increased creatinine** | 0 | 0 | 0 | 0 | 0 | 0 | 0 | 2 (40.0) | 2 (7.7) |
| **Renal failure** | 0 | 0 | 0 | 0 | 0 | 0 | 1 (16.7) | 0 | 1 (3.8) |

Abbreviations: DLT, dose-limiting toxicity.

**Table S2 PK parameters of plasma CVL218 by cohort after a single dose (Cycle 0 Day 1).**

| **Dose** | **n** | **C_max_** | **AUC_0-t_** | **AUC_0-inf_** | **Cl/F** | **V_z_/F** | **T_max_** | **T_1/2_** |
| --- | --- | --- | --- | --- | --- | --- | --- | --- |
|  |  | (ng/mL) | (Hour*ng/mL) | (Hour*ng/mL) | (mL/Hour) | (mL) | (Hour) | (Hour) |
|  |  | Geometric Mean | | | | | Median (Min, Max) | Mean |
| 100mg QD | 1 | 40.9 | 207.0 | 231.0 | 432000.0 | 2270000.0 | 2（2, 2） | 3.6 |
| 200mg QD | 1 | 218.0 | 1310.0 | 1360.0 | 147000.0 | 1080000.0 | 1（1, 1） | 5.1 |
| 400mg QD | 1 | 334.0 | 1900.0 | 2010.0 | 199000.0 | 1710000.0 | 2（2, 2） | 6.0 |
| 700mg QD | 4 | 360.0 | 3260.0 | 3450.0 | 203000.0 | 1700000.0 | 3（2, 3） | 6.3 |
| 1000mg QD | 4 | 579.5 | 4095.0 | 4820.0 | 212000.0 | 2205000.0 | 2.5（1, 4） | 6.2 |
| 1200mg QD | 4 | 547.5 | 4175.0 | 4415.0 | 276000.0 | 2155000.0 | 4（3, 4） | 6.3 |
| 1400mg QD | 6 | 633.0 | 5190.0 | 5360.0 | 274500.0 | 1920000.0 | 2.5（2, 5） | 4.7 |
| 1700mg QD | 5 | 408.0 | 4070.0 | 4180.0 | 407000.0 | 2780000.0 | 4（3, 5） | 5.8 |

Abbreviations: C_max_, peak concentration; AUC_0-t_, area under the concentration-time curve from 0 to last measurable concentration; AUC_0-inf_, area under the concentration-time curve from 0 to infinity; CL⁄F, oral clearance; Vz/F, apparent volume of distribution；T_max_, time to reach C_max_; T_1/2_, elimination half-life.

**Table S3 PK parameters of plasma CVL218 by cohort after multiple doses (Cycle 1 Day 28).**

| **Dose** | **n** | **C_max_** | **AUC_0-t_** | **AUC_0-24_** | **AUC_0-inf_** | **Cl/F** | **V_z_/F** | **T_max_** | **T_1/2_** | **C_minss_** | **C_avgss_** | **AUC_TAU** |
| --- | --- | --- | --- | --- | --- | --- | --- | --- | --- | --- | --- | --- |
|  |  | (ng/mL) | (Hour*ng/mL) | (Hour*ng/mL) | (Hour*ng/mL) | (mL/Hour) | (mL) | (Hour) | (Hour) | (ng/ml) | (ng/ml) | (Hour*ng/ml) |
|  |  | Geometric Mean | | | | | | Median (Min, Max) | Mean | | | |
| 50mg BID | 1 | 15.6 | 77.9 | 70.9 | - | 705000.0 | 6870000.0 | 1（1,1） | - | 0 | 5.91 | 70.9 |
| 100mg BID | 1 | 148.0 | 1930.0 | 822.0 | - | 122000.0 | 1750000.0 | 1（1,1） | - | 38 | 68.5 | 822 |
| 200mg BID | 1 | 156.0 | 1360.0 | 808.0 | - | 248000.0 | 4780000.0 | 2（2,2） |  | 28.6 | 67.3 | 808 |
| 350mg BID | 4 | 313.0 | 4750.0 | 2230.0 | 5860.0 | 157000.0 | 1400000.0 | 2（1,2） | 5.4 | 72.2 | 181 | 2170 |
| 500mg BID | 4 | 562.5 | 6830.0 | 3210.0 | 6920.0 | 158500.0 | 1380000.0 | 3（2,5） | 4.1 | 177 | 323 | 3870 |
| 600mg BID | 4 | 311.0 | 2560.0 | 1880.0 | 7700.0 | 318000.0 | 2440000.0 | 2（2,2） | 5.1 | 118 | 234 | 2810 |
| 700mg BID | 6 | 660.0 | 3830.0 | 3830.0 | 4170.0 | 183000.0 | 1090000.0 | 2（2,3） | 3.9 | 127 | 371 | 4450 |
| 850mg BID | 5 | 776.0 | 5150.0 | 5150.0 | 2940.0 | 230000.0 | 1532500.0 | 2（2,2） | 4.4 | 194 | 429 | 5150 |

Abbreviations: C_max_, peak concentration; AUC_0-t_, area under the concentration-time curve from 0 to last measurable concentration; AUC_0-12_, area under the concentration-time curve from 0 to 12 hours; AUC_0-inf_, area under the concentration-time curve from 0 to infinity; CL⁄F, oral clearance; Vz/F, apparent volume of distribution；T_max_, time to reach C_max_; T_1/2_, elimination half-life; C_minss_, steady-state trough concentration; C_avgss_, mean steady-state concentration; AUC_TAU, area under the concentration-time curve between one dosing interval (24 hours) at steady-state.

**Table S4 Tumor response according to tumor types.**

|  | **Lung cancer (N=10)** | **Breast cancer (N=7)** | **Other types (N=7)** |
| --- | --- | --- | --- |
| **BOR**, n (%) |  |  |  |
| CR | 0 | 0 | 0 |
| PR | 0 | 0 | 0 |
| SD | 6 (60.0) | 5 (71.4) | 6 (85.7) |
| PD | 4 (40.0) | 2 (28.6) | 1 (14.3) |
| **ORR**, n (%)  (95%CI) | 0  (0, 30.9) | 0  (0, 41.0) | 0  (0, 41) |
| **DCR**, n (%)  (95%CI) | 60  (26.2, 87.8) | 71.4  (29.0, 96.3) | 85.7  (42.1, 99.6) |
| **mPFS**  **(months)** | 2.12 | 1.71 | 1.91 |

Abbreviations: BOR, best overall response; CR, complete response; PR, partial response; SD, stable disease; PD, progressive disease; ORR, objective response rate; DCR, disease control rate; mPFS, median progression free survival.
